# From immune evasion to broad *in silico* binding: computational optimization of SARS-CoV-2 RBD-targeting nanobody

**DOI:** 10.3389/fimmu.2025.1637955

**Published:** 2025-08-14

**Authors:** Shuyuan Cao, Bo Sun, Feng Gao

**Affiliations:** ^1^ Department of Physics, School of Science, Tianjin University, Tianjin, China; ^2^ State Key Laboratory of Synthetic Biology, Tianjin University, Tianjin, China; ^3^ Frontiers Science Center for Synthetic Biology and Key Laboratory of Systems Bioengineering (Ministry of Education), Tianjin University, Tianjin, China

**Keywords:** SARS-CoV-2, immune evasion, nanobody, computational pipeline, affinity maturation

## Abstract

**Introduction:**

The rapid evolution of SARS-CoV-2 Omicron variants highlights the urgent need for therapeutic strategies that can target viral evolution and leverage host immune recognition mechanisms. This study uses molecular dynamics (MD) simulations to analyze the immune evasion mechanisms of class 1 nanobodies against emerging SARS-CoV-2 variants, and to develop an efficient *in silico* pipeline for rapid affinity optimization.

**Methods:**

We employed MD simulations and binding free energy calculations to investigate the immune evasion mechanisms of four class 1 nanobodies (R14, DL4, V_H_ ab6, and Nanosota9) against wild-type (WT) and Omicron variants, including BA.2, JN.1, and KP.3/XEC. Building on these findings, we established a streamlined nanobody optimization pipeline integrating high-throughput mutagenesis of complementarity-determining regions (CDRs) and hotspot residues, protein-protein docking, and MD simulations.

**Results:**

MD analysis confirmed that the immune evasion mechanism of KP.3/XEC is significantly associated with the Q493E mutation, which weakens electrostatic interactions between the nanobodies and the receptor binding domain (RBD). Through our pipeline, we identified high-affinity mutants including 3 for R14, 3 for DL4, 11 for VH ab6, and 9 for Nanosota9. The optimized R14 variant L29W/S52C/A101V demonstrated exceptional performance, achieving a 62.6% binding energy improvement against JN.1 (-76.88 kcal/mol compared to -47.3 kcal/mol for original R14 nanobody) while maintaining < 15% affinity variation across variants (compared to > 40% for original R14 nanobody).

**Discussion:**

This study demonstrates that in silico affinity enhancement is a rapid and resource-efficient approach to repurpose nanobodies against SARS-CoV-2 variants, significantly accelerating affinity optimization while reducing experimental demands. This computational approach expedites the optimization of nanobody binding affinities while minimizing experimental resource requirements. By enhancing nanobody efficacy, our method provides a viable framework for developing targeted countermeasures against evolving SARS-CoV-2 variants and other pathogens.

## Graphical Abstract

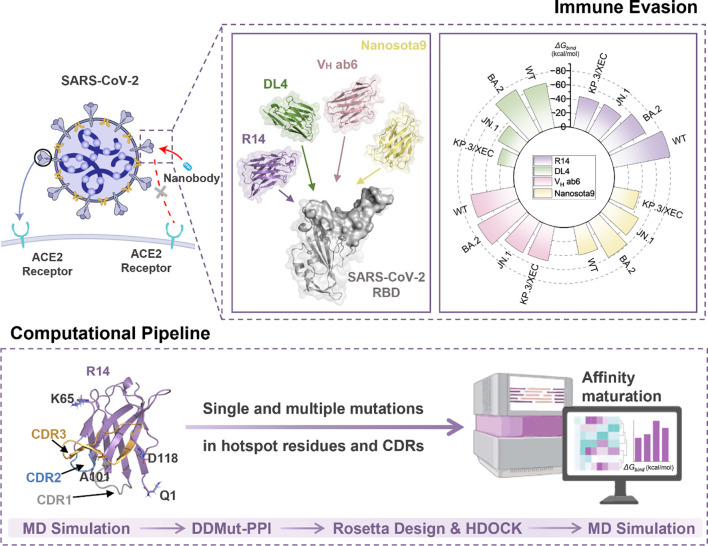

## 1 Introduction

Severe acute respiratory syndrome coronavirus 2 (SARS-CoV-2) continues to evolve with sustained global transmission, posing significant challenges to public health (1, 2). The virus relies on its spike glycoprotein (S protein) to bind with the angiotensin-converting enzyme 2 (ACE2) receptor on host cells, a critical step in viral entry (2–5). This interaction represents a critical target for therapeutic interventions, including monoclonal antibodies and antiviral drugs (6, 7). However, the rapid emergence of new variants carrying mutations in the receptor binding domain (RBD) of the S protein has raised concerns about the effectiveness of existing treatments and vaccines (1, 8, 9).

Notably, recent Omicron subvariants, including JN.1, KP.3, and XEC, exhibit enhanced transmissibility and immune evasion capabilities. The JN.1 variant, which first appeared in early 2024, has spread rapidly around the world, showcasing mutations that enhance its transmissibility. Specifically, JN.1 carries critical RBD mutations (R346T and F456L) that strengthen ACE2 receptor binding affinity and promote immune escape (1, 10, 11). Similarly, the KP.3 variant, a sublineage of JN.1, presents additional alterations in its RBD, particularly Q493E, which may further enhance its ability to evade neutralizing antibodies (1, 11). Meanwhile, the XEC variant, a recombinant of JN.1 subvariants KS.1 and KP.3.3, demonstrates a growth advantage in the population, suggesting enhanced virulence, greater immune evasion and caused breakthrough infectivity (12, 13). The World Health Organization (WHO) has classified JN.1 as a Variant of Interest (VOI) and KP.3 and XEC as Variants Under Monitoring (VUM), highlighting their potential global impact (14).

Nanobodies (Nbs), small single-domain antibodies derived from camelids, represent a promising therapeutic approach against SARS-CoV-2 (15). Structurally, nanobodies are composed of four highly conserved framework regions (FRs) interspersed with three hypervariable regions known as complementarity-determining regions (CDRs). These CDRs are responsible for antigen recognition and binding specificity (16, 17), making them critical for targeting viral epitopes. Compared to conventional monoclonal antibodies, nanobodies exhibited superior stability, simpler production processes, and enhanced capacity to recognize conformational epitopes properties (17) that make them particularly suitable for targeting the RBD of the S protein. Additionally, studies have shown that aerosol delivery achieves over 80% pulmonary deposition efficiency of nanobodies in murine models, ensuring localized antiviral activity at efficacious doses without systemic toxicity (18, 19). This delivery method enables direct lung targeting, maximizing local drug concentrations while minimizing systemic exposure. However, a critical limitation persists: most existing therapeutic antibodies, including nanobodies, were developed against early SARS-CoV-2 variants. The continuous accumulation of RBD mutations (17, 20) poses substantial challenges for both current treatment efficacy and future therapeutic development.

Conventional nanobody discovery methods, such as phage display and immunization of camelids, present significant limitations in terms of time investment and scalability (21). These techniques typically require months to years of experimental screening and optimization, compounded by challenges in reproducibility and large-scale production (21–23). The 12-month half-life of SARS-CoV-2 antibody efficacy reported in 2024 virological surveys underscores the imperative to shorten discovery timelines (24). In contrast, computational design and optimization strategies offer a cost-effective and rapid alternative. By leveraging methods such as *ab initio* modeling, *in silico* mutagenesis, and machine learning, researchers can rapidly generate high-affinity binders with improved stability and solubility (25–28). This approach not only accelerates the discovery of novel nanobodies but also enables the repurposing of existing ones to target emerging variants.

This study evaluates four class 1 (29) nanobodies [R14 (30), DL4 (31), V_H_ ab6 (32), and Nanosota9 (33)], which were sequentially recognized as therapeutic candidates against evolving SARS-CoV-2 variants. Among them, V_H_ ab6 and Nanosota9 have been recognized as broad-spectrum nanobodies because they demonstrated cross-variant neutralization activity. We systematically investigate their binding mechanisms across SARS-CoV-2 wild-type (WT) and variants, including BA.2, JN.1, and KP.3/XEC. Through detailed energetic and conformational analyses to elucidate the impact of viral mutations on binding affinity.

**Figure 1:**
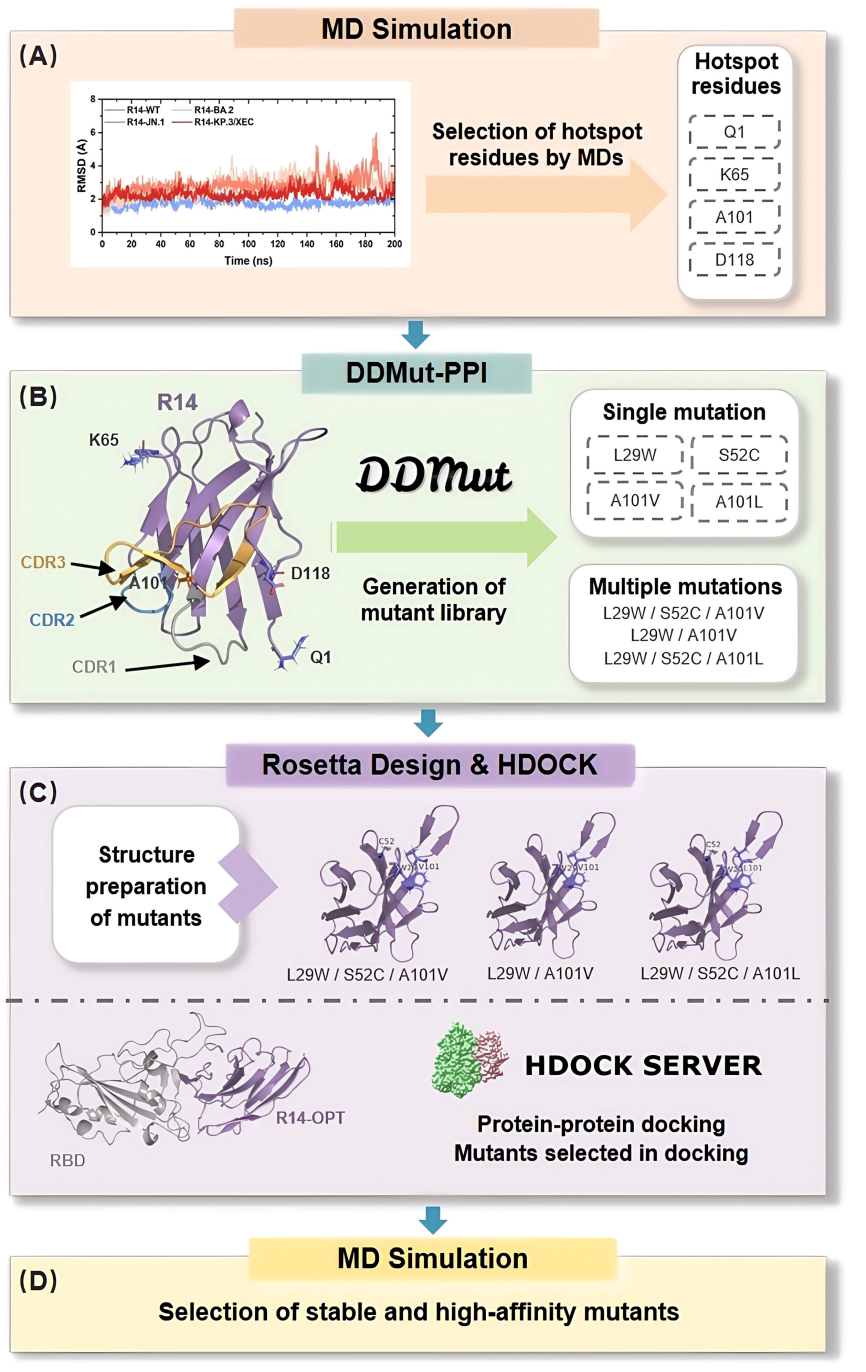
*In Silico* Affinity Maturation Pipeline for Optimizing Nanobody Binding to Omicron RBD. The process begins with preliminary analysis of the nanobody-RBD affinity through MD simulations, focusing on the selection of hotspot residues **(A)**. Next, DDMut-PPI is utilized to generate a virtual library by applying single mutation in hotspot residues and CDRs, as well as multiple mutations to identify beneficial mutation combinations **(B)**. Structure preparation of the mutants is performed using Rosetta Design **(C)**. Following this, protein-protein docking is conducted using HDOCK, where binding score is employed to select suitable mutants. Finally, MD simulations are used to select high-affinity and stable mutants, to validate the previous steps **(D)**.

Furthermore, the development of nanobodies typically requires experimental screening of large antibody libraries, which is not only costly and time-consuming but also faces challenges such as low expression, poor solubility, and multispecificity (21–23). These persistent limitations underscore the critical need for both innovative development approaches and efficient repurposing strategies for existing nanobodies (34, 35). To address these challenges, we present a streamlined computational pipeline (**Figure 1**) that generates a library of high-affinity and stable mutants by targeting substitutions in the CDRs and hotspot residues. The crystal structure of the nanobody in complex with the SARS-CoV-2 RBD served as template for high-throughput computational mutagenesis. High-affinity mutants were selected through protein-protein docking and molecular dynamics (MD) simulations, enabling the identification of favorable mutations with enhanced binding properties. This approach provides a robust framework for the development of effective therapeutic agents against SARS-CoV-2 and its evolving variants.

## 2 Methods

### 2.1 System preparation

The crystal structures of four SARS-CoV-2 RBD targeting nanobodies (30–33) R14 (PDB ID: 7WD1) (30), DL4 (PDB ID: 7F5G) (31), V_H_ ab6 (PDB ID: 8DLX) (32), and Nanosota9 (PDB ID:9CO9) (33) and SARS-CoV-2 WT (PDB ID: 7WD1) (30), BA.2 (PDB ID: 7ZF8) (36), JN.1 (PDB ID: 8Y5J) (37) RBDs were obtained from the RCSB Protein Data Bank (38). The KP.3/XEC RBD structure was generated by computational mutagenesis using RosettaDesign (39) with JN.1 RBD as template. These nanobodies were subsequently docked with the RBDs using the HDOCK server (40), and favorable binding conformations were selected based on docking score and confidence score (>0.99).

### 2.2 Molecular dynamics simulation

MD simulation was conducted using GROMACS 2022.5 with the CHARMM36 force field (41). The complex solvated in a cubic box using the TIP3P water model, and counter ions were added to neutralize the protein in the aqueous system. Energy minimization was executed through the steepest descent algorithm over 50,000 iterations to alleviate any high-energy contacts. Then, a two-step equilibration process was performed for 100 ps. First, canonical ensemble (NVT, constant number of particles, volume, and temperature) equilibration was conducted, maintaining the temperature at 310.15 K using the v-rescale thermostat, followed by isothermal-isobaric ensemble (NPT, constant number of particles, pressure, and temperature) equilibration with the c-rescale method for isobaric coupling at a reference pressure of 1.0 bar. Throughout the simulation, periodic boundary conditions (PBC) were applied, and long-range electrostatic interactions were calculated using the Particle Mesh Ewald (PME) method with a cutoff radius of 12 Å. Finally, a 200 ns production MD run was performed on the optimized system, with a time step of 2 fs, recording energy and log data every 1000 steps. The MD trajectory was analyzed to calculate the root mean square deviation (RMSD) and the number of hydrogen bonds, providing insights into the stability and interactions of the complexes.

### 2.3 Binding free energy calculations

Binding free energy calculations were performed using the Molecular Mechanics/Poisson-Boltzmann (Generalized Born) Surface Area (MM/PB(GB)SA) method. To ensure robust sampling, equilibrium trajectory data were extracted from the production phase of the MD simulations. Specifically, for each system, the last 50 ns of equilibrium trajectory data were used, with snapshots collected at 50 ps intervals. This methodology provided a total of 1,000 frames for binding free energy calculations.

The binding free energy ( $\text{Δ}G_{bind}$ ) of the Nb-RBD complex can be regarded as the sum of enthalpy term (Δ*H*) and entropy term (-*T*Δ*S*):


$$\begin{array}{c} \text{Δ}G_{bind}=\text{Δ}H-T\Delta S\; \end{array}$$

In this context, the enthalpy change is composed of ensemble average interaction energy $\left(E\right)$ and solvation energy ( $\text{Δ}G_{sol}$ ), in which $\left(E\right)$ can be separated into the electrostatic interaction energy (Δ*E_ele_*) and van der Waals interaction energy (Δ*E_vdW_*).


$$\text{Δ}H=\langle E\rangle +\text{Δ}G_{sol}=\text{Δ}E_{ele}+\Delta E_{vdW}+\text{Δ}G_{sol}$$

The solvation energy ( $\text{Δ}G_{sol}$ ) is further divided into polar solvation energy ( $\text{Δ}G_{pb}$ ) and nonpolar solvation energy ( $\text{Δ}G_{SA}$ ):


$$\begin{array}{c} \text{Δ}G_{sol}=\text{Δ}G_{pb/gb}-\text{Δ}G_{SA}\; \end{array}$$

In this study, a single trajectory approach was adopted due to its lower noise level and its suitability for systems with minimal structural rearrangement during binding. The entropy contribution was generally excluded from the calculations (42), as entropy differences associated with relative binding affinities were expected to be small, with only minor variations arising from mutations. MM/PB(GB)SA calculations were performed using gmx_MMPBSA version 1.6.3 (43), a novel tool developed for endpoint free energy calculations from MD trajectories.

To further elucidate the binding interactions, an energy decomposition analysis was conducted to assess the contribution of each residue to the binding free energy. This analysis highlights key residues that stabilize or destabilize the Nb-RBD complex, identifying potential targets for therapeutic intervention.

### 2.4 Generation of mutant library

The HDOCK generated Nb-RBD complexes were subjected to single-point mutations using DDMut-PPI (44). Single-point mutations were introduced at CDRs and hotspot residues, substituting each residue with the 19 alternative amino acids, excluding itself. The resulting changes in binding affinities were calculated as ΔΔ*G* (ΔΔ*G*=Δ*G_WT_*-Δ*G_mutant_*, kcal/mol), with ΔΔ*G* > 0 indicating increased affinity and ΔΔ*G* < 0 indicating decreased affinity compared to the wild type.

Empirical evidence and previous studies indicate that combinations of individually beneficial mutations are more likely to result in additive or synergistic improvements in binding affinity (45, 46). Therefore, we prioritized the combination of single-point mutations with ΔΔ*G* > 0 for subsequent analysis. Stabilizing mutations (ΔΔ*G* > 0) identified across variant complexes were subsequently combined for multiple mutations analysis using DDMut-PPI’s combinatorial module. Utilizing this approach, an *in silico* library comprising high-binding and stable single-point and multiple-points mutations in the nanobodies R14, DL4, V_H_ ab6, and Nanosota9 were generated.

### 2.5 Interaction assessment and selection of best mutants

To validate our streamlined computational pipeline, mutations identified through computational affinity maturation were introduced into R14. The resulting mutant structures were docked with the RBD of the KP.3/XEC variant using HDOCK, employing its scoring function to filter for the best-performing mutants. The evaluation focused on predicted binding affinities and structural compatibility with the RBD.

Subsequently, the optimized combinations for R14 were subjected to a 200 ns production MD simulation with WT and three variants (BA.2, JN.1, and KP.3/XEC) complexes. The dynamic stability and interaction profiles of the mutants were analyzed using RMSD and MM/PB(GB)SA method. RMSD was utilized to assess conformational changes in the protein backbone, while MM/PB(GB)SA was employed to calculate binding free energies. These analyses enabled the identification of high-affinity and stable mutants, further refining the selection based on dynamic stability and interaction profiles. This approach ensured a robust assessment of the best-performing mutants.

## 3 Results

### 3.1 Anti-SARS-CoV-2 Omicron variants spectrum of nanobodies

**Figure 2:**
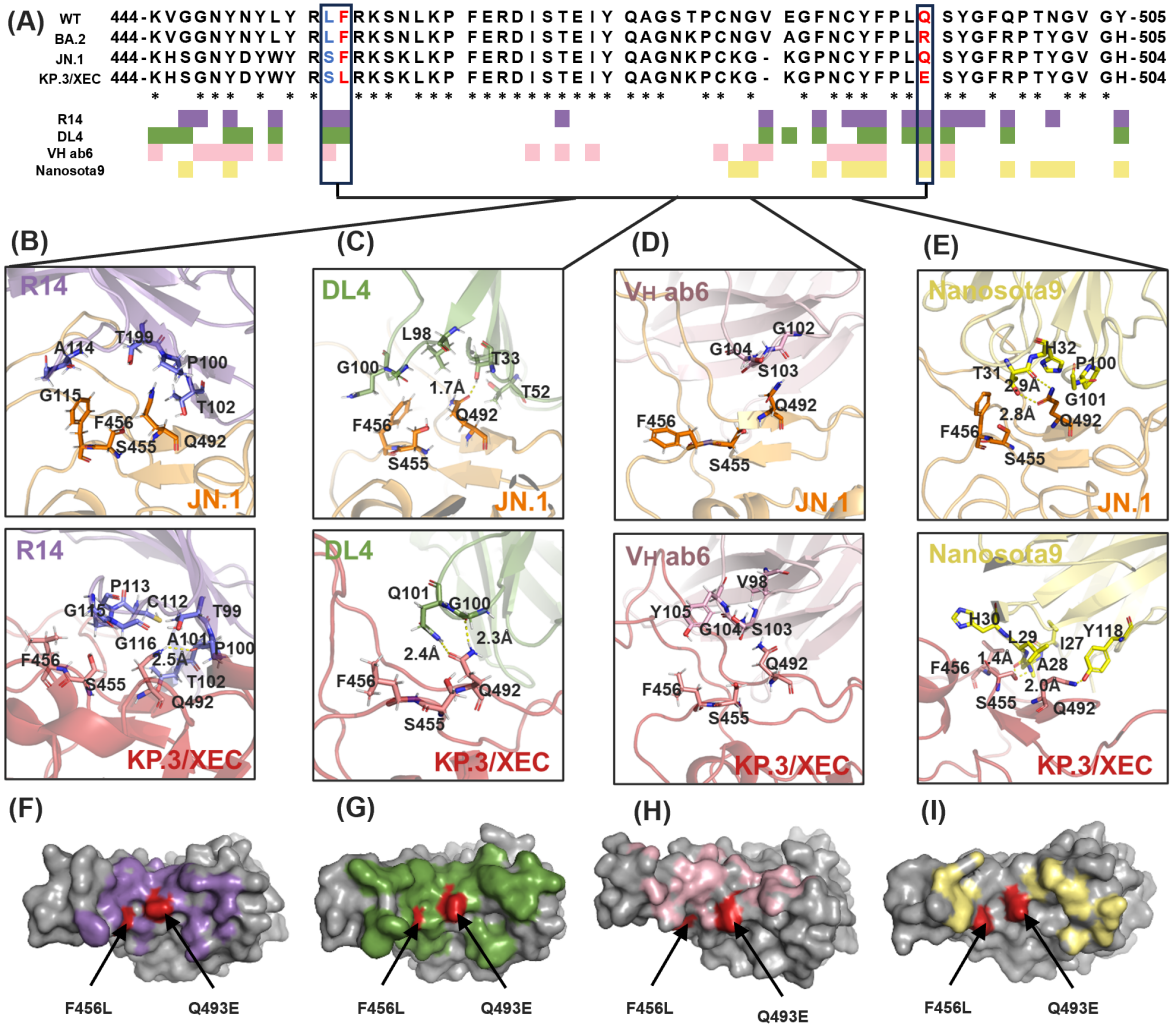
Evolution of Nb binding epitopes within the Omicron RBD. **(A)** Sequence alignment and binding epitopes of Nb-RBD residues among three Omicron subvariants: BA.2, JN.1, and KP.3/XEC. RBD residues in direct contact with R14, DL4, V_H_ ab6, and Nanosota9 are colored purple, green, pink, and yellow, respectively. RBD residues that underwent mutations in the JN.1 and KP.3/XEC subvariants are highlighted in bold blue and bold red, and they are boxed for emphasis. Asterisk indicated positions with a single, fully conserved residue. **(B-E)** Structural details of Nb-RBD complex that underwent mutations between the Omicron subvariants JN.1 and KP.3/XEC. **(F-I)** Mapping of RBD residues that underwent mutations in the Omicron subvariant KP.3/XEC. RBD surface in direct contact with R14, DL4, V_H_ ab6, and Nanosota9 are colored purple, green, pink, and yellow, respectively.

In this study, we evaluated the antiviral spectrum of four RBD-targeting nanobodies (R14, DL4, V_H_ ab6, and Nanosota9) against SARS-CoV-2 Omicron variants. Despite the RBD accumulating mutations at 3–5 times the rate of other spike protein domains (47), RBD-targeting antibodies remain clinically dominant due to their direct disruption of ACE2 binding (48). To elucidate their binding characteristics, we conducted a sequence alignment of RBDs from BA.2, JN.1, and KP.3/XEC. The analysis identified three critical mutations (L455S, F456L, and Q493E) within the RBD regions that directly interact with the nanobodies. Epitope mapping (**Figure 2A**) revealed that V_H_ ab6 and Nanosota9 target relatively conserved epitopes on the Omicron RBD, whereas R14 and DL4 bind to more variable regions. These findings indicate that the binding affinities of V_H_ ab6 and Nanosota9 exhibit minimal variability across different Omicron subvariants, enabling effective neutralization of the most predominant variants (**Figures 2F-I**).

Furthermore, structural analysis revealed that R14 primarily interacts with KP.3/XEC through main-chain functional groups, suggesting that side-chain variations exert minimal influence on binding (**Figure 2B**). This finding accounts for the partial neutralization efficacy of R14 against KP.3/XEC. In contrast, V_H_ ab6 and Nanosota9 involve changes in both main-chain and side-chain interactions, leading to a more pronounced influence of side-chain groups on overall antibody affinity (**Figures 2D, E**). These structural insights align with biochemical and virological findings regarding the antiviral efficacy of each nanobody against earlier and recent Omicron subvariants.

### 3.2 Immune escape mechanisms of SARS-CoV-2 Omicron variants against nanobodies

To further investigate the interactions between nanobodies and SARS-CoV-2 Omicron variants, we performed MD simulations on sixteen systems, including four nanobodies (R14, DL4, V_H_ ab6, and Nanosota9) and four RBDs (WT, BA.2, JN.1, and KP.3/XEC). We assigned the complexes formed by R14 with these RBDs as R14-RBD systems (R14-WT, R14-BA.2, R14-JN.1 and R14-KP.3/XEC). The naming convention for the complexes formed by the other nanobodies followed the same pattern. Throughout the simulations, all systems maintained stable binding states, with RMSD fluctuations consistently below 6 Å (**Supplementary Figures S1A-D**). Based on the equilibrium portion of the MD trajectory (150–200 ns), the free energy landscape (FEL) analysis (**Supplementary Figure S2**) was performed to explore the distribution of conformations and stability of Nb-RBD systems. In general, lower energy states indicate the corresponding conformations possess greater stability. Notably, the complexes formed between JN.1 and KP.3/XEC RBDs and the nanobodies exhibited increased flexibility at the binding interface compared to the complexes formed between the WT and BA.2 RBDs and the nanobodies. This enhanced flexibility suggests more dynamic interactions, potentially affecting binding characteristics and antibody efficacy.

**Figure 3:**
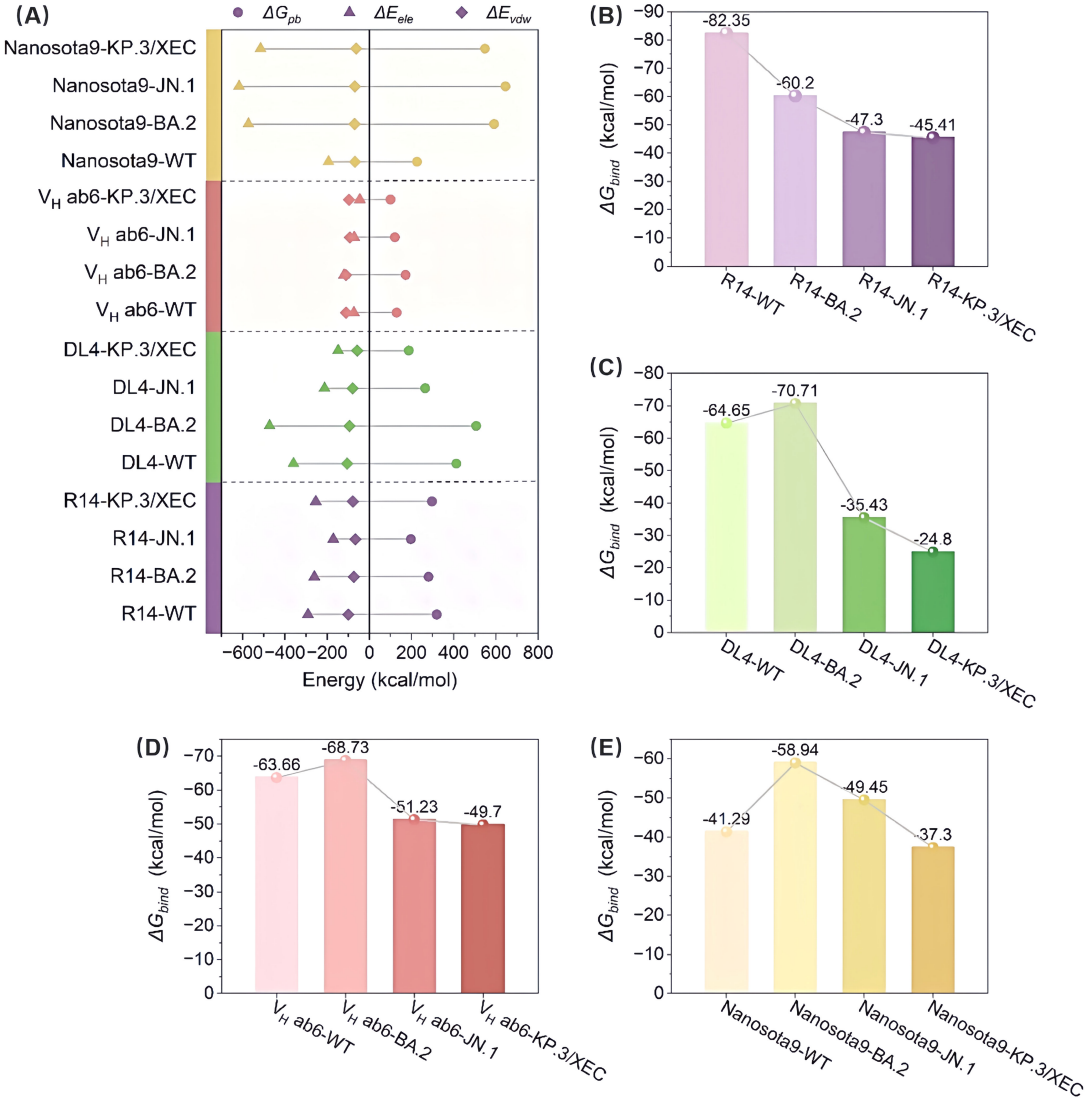
Evaluation of the binding affinity of the Nb-RBD complexes by MM/PBSA. **(A)** Shows the contributions to the binding free energy, including *van der Waals* interactions (Δ*E_vdw_*), electrostatic interactions (Δ*E_ele_*), and polar solvation free energy (Δ*G_pb_*), for R14, DL4, V_H_ ab6, and Nanosota9 with four different RBDs (WT, BA.2, JN.1, and KP.3/XEC). **(B-E)** The total binding free energy (Δ*G_bind_*) for the corresponding complexes.

To assess the effect of mutations on binding modes and energy changes, we calculated the binding free energies using the MM/PBSA method. The binding affinities of R14 and DL4 were significantly reduced, with the R14-KP.3/XEC having a binding affinity of -45.41 kcal/mol, representing increases of 36.94 kcal/mol and 39.85 kcal/mol, respectively, compared to their WT systems (**Figures 3B, C**). In contrast, the reduction in binding affinity for V_H_ ab6 and Nanosota9 was more gradual, with increases of 13.96 kcal/mol and 3.99 kcal/mol, respectively, compared to their WT complexes, consistent with the analysis in section 3.1 regarding the antiviral spectrum of nanobodies (**Figures 3D, E**). These findings were further validated through MM/GBSA cross-verification (**Supplementary Table S1**), confirming the robustness of our predictions. Although the absolute binding energies varied between methods due to differences in the solvation model, both approaches showed consistent trends in changes to binding affinity across variants.

Overall, the binding affinities of JN.1 and KP.3/XEC variants to nanobodies were markedly lower than those of WT and BA.2 variants. This discrepancy primarily arises from a significant reduction in electrostatic and *van der Waals* (*vdW*) energies (**Figure 3A**). The former is attributed to the introduction of positively charged residues by mutations, while the latter results from unstable binding modes between the mutated RBD and nanobodies, leading to fewer atomic contacts. In addition, hydrogen bond network analysis (**Supplementary Figure S3**) revealed that the JN.1 and KP.3/XEC variants had more low-occupancy hydrogen bonds (occupancy < 70%) and fewer high-occupancy hydrogen bonds (occupancy ≥ 70%), which further impacted the stability of the complex.

**Figure 4:**
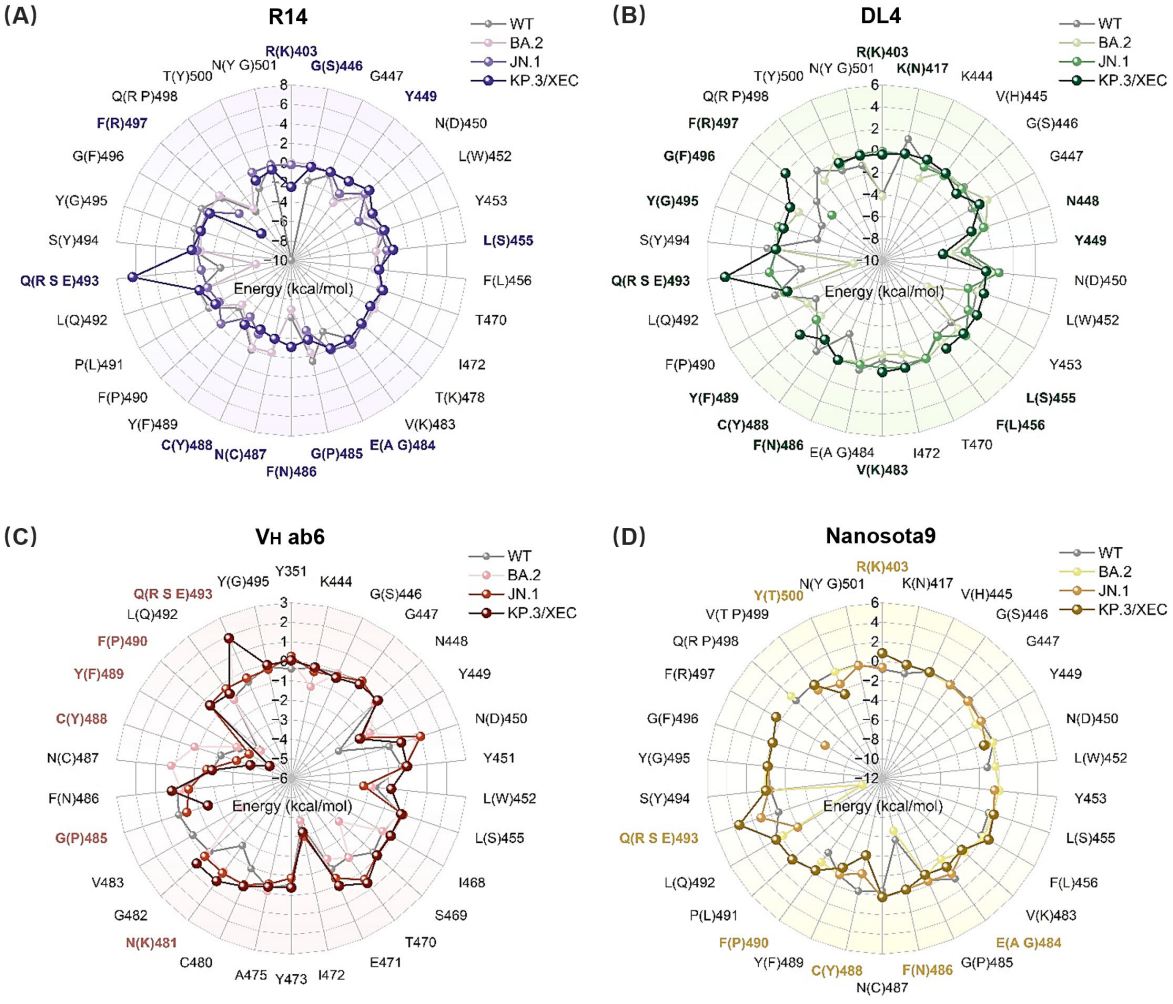
Binding free energy contributions of key residues in RBD. **(A)** R14, **(B)** DL4, **(C)** V_H_ ab6, and **(D)** Nanosota9 interacting with RBD (WT, BA.2, JN.1, and KP.3/XEC). Residues with energy differences (|ΔΔ*G_Var_*_–_ *_WT_* | ≥ 1 kcal/mol) between mutant and WT systems are highlighted in bold purple, green, pink, and yellow, respectively.

Subsequently, we calculated the binding free energy contributions of residues near the binding interface using residue decomposition methods. Residues with an absolute binding free energy difference ≥ 1 kcal/mol compared to the WT system were defined as hotspot residues. In the R14-KP.3/XEC complex, a greater number of hotspot residues were identified than in the R14-JN.1 complex (**Figure 4A**, **Supplementary Figures S4A, B**), likely due to the unique F456L and Q493E mutations in KP.3/XEC. Notably, the Q493E mutation in the R14-KP.3/XEC complex exhibited a substantial energy change, attributed to the transformation from neutral glutamine to negatively charged glutamate at physiological pH, enhancing electrostatic repulsion between nanobody and RBD. This mutation also disrupted existing hydrogen bonds, further destabilizing the complex (**Supplementary Figure S4**). Similarly, the charged mutation at residue 493 significantly decreased electrostatic interactions in the DL4, V_H_ ab6, and Nanosota9 systems (**Figure 3A**). In the DL4-KP.3/XEC complex, residues F489, G495, and R497 exhibited notable energy reductions, clustering in a similar region, suggesting substantial conformational changes. The V_H_ ab6 and Nanosota9 complexes displayed comparable binding modes, primarily influenced by the Q493E mutation in KP.3/XEC (**Figures 4C, D**, **Supplementary Figures S4E-H**). However, the charged residues introduced by the mutations did not approach the binding interface closely enough to significantly impact binding affinity compared to R14 and DL4.

### 3.3 Generation of mutant library and assessment of binding affinity

**Figure 5:**
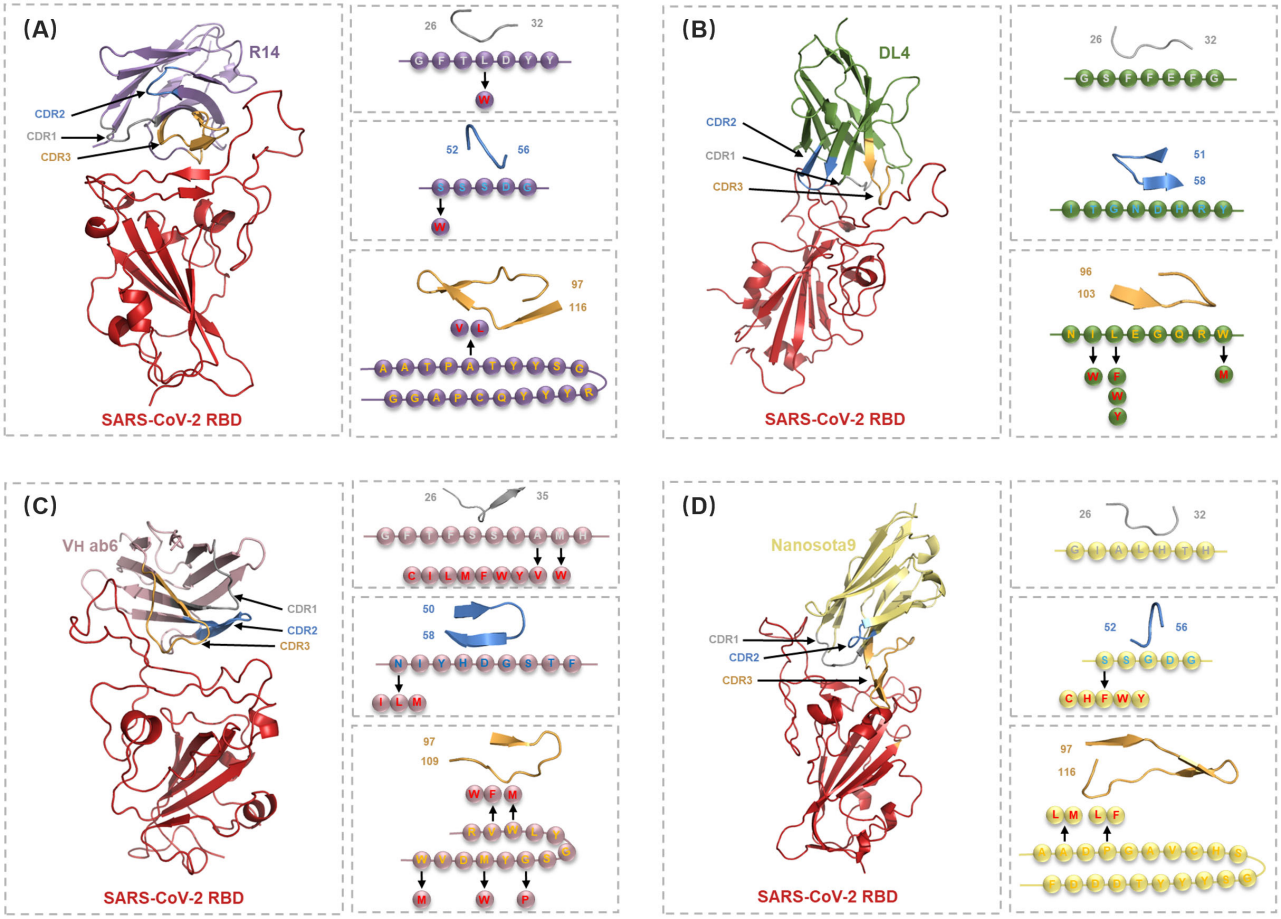
Detailed structural analysis of Nb-RBD complex and selection of residues for the *in silico* mutagenesis. **(A-D)** Cartoon representations of the nanobodies R14 (purple), DL4 (green), V_H_ ab6 (pink), and Nanosota9 (yellow) in complex with RBD (red), illustrating the positions of CDR1 (gray), CDR2 (blue), and CDR3 (orange). Arrows indicate the mutations selected in circles within specific CDRs or hotspot residues, identified through DDMut-PPI single mutations.

The sixteen nanobody-RBD systems were analyzed using DDMut-PPI to predict high-affinity single-point mutations targeting CDRs and hotspot residues (**Supplementary Tables S2**-**3**). This computational affinity maturation approach generated a virtual library containing 2,508 mutations for R14, 1,824 for DL4, 2,432 for V_H_ ab6, and 2,508 for Nanosota9. Screening for mutations with predicted ΔΔ*G* > 0 (indicating enhanced affinity and stability, **Supplementary Figure S6**) identified 4 beneficial mutations for R14, 5 for DL4, 15 for V_H_ ab6, and 9 for Nanosota9 (**Figure 5**, **Supplementary Table S4**).

Moreover, mutations within specific CDRs and hotspot residues not only alter the binding characteristics at the mutation sites but also affect the binding modes of other CDRs and framework regions. Analysis of combinatorial mutations revealed substantial variations in binding energy, with the multi-points mutant library containing 28 high-affinity variants for R14, 40 for DL4, 13,748 for V_H_ ab6, and 176 for Nanosota9. Notably, V_H_ ab6 displayed markedly greater mutational plasticity, accommodating significantly more viable multi-points mutation than other nanobodies while maintaining stable binding conformations.

### 3.4 Selection of high-affinity mutants using docking and MD simulation

High-affinity substitution mutations identified through preliminary screening using DDMut-PPI, including both single and multiple mutations, were introduced into the nanobodies (**Supplementary Table S5**). For R14 variants, docking against four SARS-CoV-2 variant RBDs using HDOCK identified three top-ranking mutation combinations exhibiting enhanced interaction (**Supplementary Table S6**).

To validate the feasibility of this nanobody repurposing pipeline, we conducted molecular dynamics simulations on the complexes of three optimized variants of R14 with four different SARS-CoV-2 variants to assess structural stability and energy changes following mutation. The RMSD was utilized to determine the conformational changes occurring in the protein backbone throughout the simulation, indicating the dynamic stability of the complex. Although the RMSD of the optimized structure was higher than that of the initial structure, reflecting internal structural changes that occurred during the optimization, it remained stable throughout the simulation. The RMSD fluctuations of RBD and R14 mutated nanobody complex remained consistently below 6 Å (**Supplementary Figure S6**), induced by minimal fluctuations and suggesting stable binding. The increased RMSD post-optimization, while stable, indicates a reorganization of the protein structure that may enhance the interaction with the target, thus aligning with the improved binding energy observed in the analysis.

**Figure 6:**
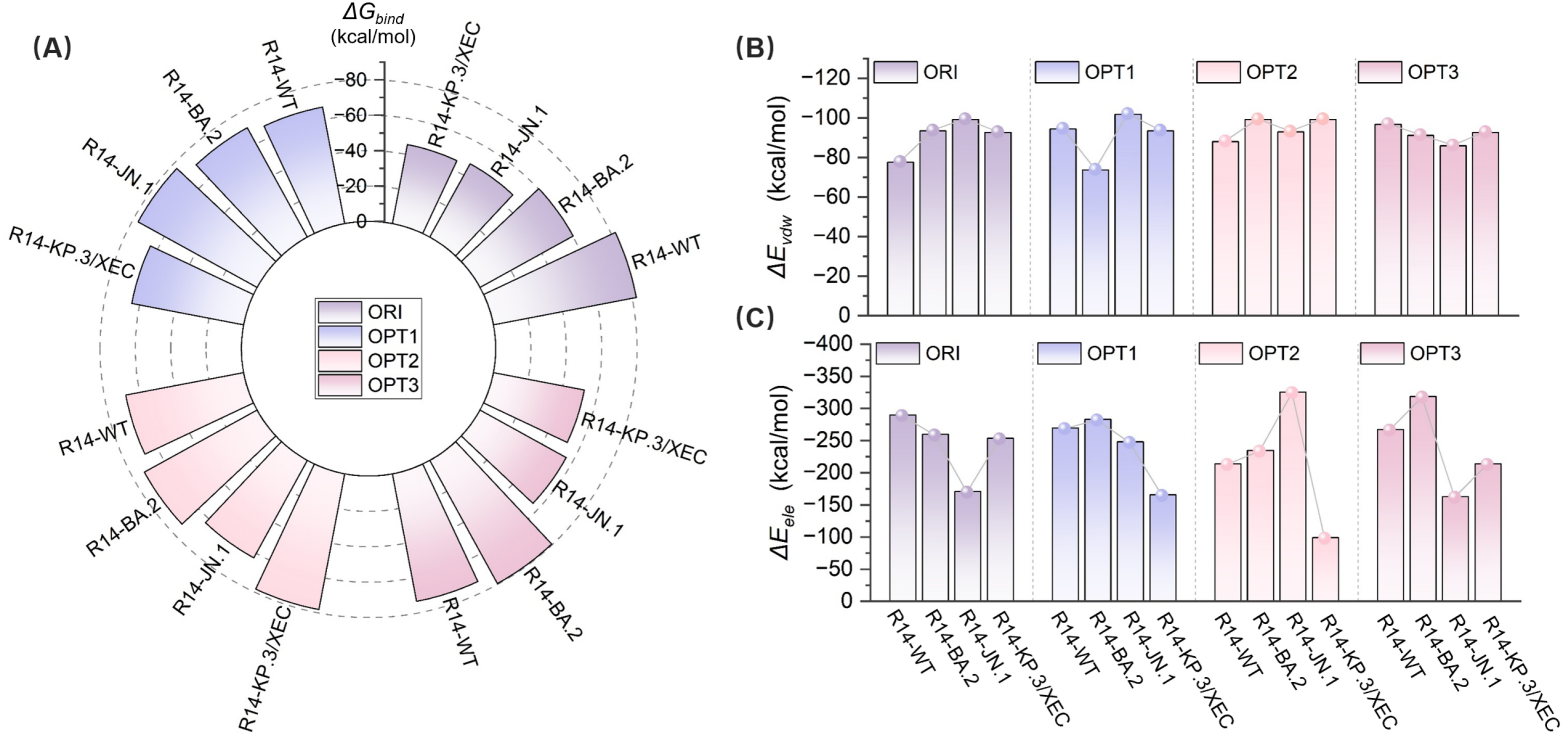
Evaluation of the binding affinity of original and optimized R14- RBD complexes by MM-PBSA. **(A)** Total binding free energy (Δ*G_bind_*) for WT, BA.2, JN.1, and KP.3/XEC variants with original (ORI) and optimized (OPT1, OPT2, OPT3) nanobodies. **(B)** *van der Waals* interaction energies (Δ*E_vdW_*) for the complexes. **(C)** Electrostatic interaction energies (Δ*E_ele_*) for the complexes.

Binding affinity serves as a key metric for evaluating the strength and specificity of nanobody-antigen interactions. We designate the complexes formed by the original R14 nanobody and its three optimized R14 as R14-ORI, R14-OPT1 (L29W/S52C/A101V), R14-OPT2 (L29W/A101V), and R14-OPT3 (L29W/S52C/A101L), respectively. Comprehensive binding free energy analysis (**Figure 6**) revealed substantial improvements in binding affinity against all SARS-CoV-2 variants mentioned above in this study, following nanobody optimization. The R14-ORI exhibited progressively weaker binding from WT (-82.35 kcal/mol) to KP.3/XEC (-45.41 kcal/mol), consistent with evolving immune evasion. Optimized Nb-RBD complexes demonstrated markedly enhanced and more uniform affinities. R14-OPT1 showed particularly strong JN.1 binding affinity, with a free energy of -76.88 kcal/mol compared to -47.3 kcal/mol for R14-ORI, while maintaining stable WT interactions at -67.04 kcal/mol. R14-OPT2 achieved exceptional KP.3/XEC binding affinity at -78.09 kcal/mol with consistent performance across variants. R14-OPT3 displayed optimal BA.2 affinity at -79.47 kcal/mol despite a moderate reduction for JN.1 to -55.69 kcal/mol. Notably, the binding free energy for the R14-OPT2-KP.3/XEC system exhibited slight discrepancies compared to the DDMut-PPI and HDOCK predictions, which can be attributed to the stronger stability of this system, resulting in lower overall perturbations in its structure compared to other similar systems.

Furthermore, R14-OPT1 exhibited the most balanced binding characteristics, with affinities ranging from -64.49 kcal/mol (KP.3/XEC) to -76.88 kcal/mol (JN.1), a variation of 16.1% compared to the 44.9% variation observed for R14-ORI (-45.41 to -82.35 kcal/mol). These energy landscapes suggest R14-OPT1 represent the optimal compromise between variant coverage and binding potency, particularly given its superior JN.1 recognition (-76.88 kcal/mol versus -47.3 kcal/mol for R14-ORI). Overall, the binding affinities of the optimized R14 nanobody with these four variants were comparatively favorable. These findings highlight the potential of integrating computational methods for affinity maturation in the development of effective therapeutics against SARS-CoV-2.

## 4 Discussion

When facing Omicron variants, countermeasures, such as vaccines and therapeutic drugs, display weaker or even lost effectiveness (1, 8, 9). Our integrated computational analyses reveal that the Q493E mutation in emerging SARS-CoV-2 variant KP.3/XEC drives immune evasion through electrostatic disruption at nanobody-RBD interfaces. This immune evasion effect is particularly pronounced for nanobodies like R14 and DL4, which engage variable epitopes, whereas V_H_ ab6 and Nanosota9 maintain broader efficacy by targeting evolutionarily constrained regions. Critically, the structural and computational research of this part is consistent with the existing experimental studies (30–33), which also confirms the feasibility of our next workflow. Building on these mechanistic insights, we developed an efficient computational pipeline that synergistically combines integrates high-throughput mutagenesis, protein-protein docking, and MD simulations to engineer optimized nanobody variants. Unlike conventional strategies focusing exclusively on single-point mutations in either CDRs or hotspot residues, our approach comprehensively targets both regions while incorporating structural dynamics from molecular simulations (7, 28, 49, 50). This pipeline specifically targets substitutions in the CDRs and hotspot residues, generating a mutant library of high-affinity and stable mutants. This integrated methodology generates mutant libraries enriched for high-affinity, stable nanobodies, ultimately providing atomistic insights to accelerate structure-guided nanobody design and therapeutic development.

Notably, R14 emerged as the primary candidate for optimization due to its unique therapeutic and structural properties. Aerosolized R14 maintained neutralizing activity and prevented infection (30), and it exhibited exceptional conformational stability across variants (RMSD <6 Å). This resilience stems from its predominant reliance on main-chain interactions with RBD, minimizing vulnerability to side-chain mutations. Residue decomp energetics revealed that Q493E-induced repulsion is a quantifiable liability (ΔΔ*G* = +8 kcal/mol), offering a clear strategy for compensatory engineering through mutations.

The substantial binding affinity improvements achieved through computational maturation (e.g., R14-OPT1’s 62.6% ΔΔ*G* enhancement against JN.1) hold significant implications for neutralization potency. Empirical calibrations indicate that, under typical Cheng–Prusoff conditions, every ~1.4 kcal/mol reduction in Δ*G_bind_* corresponds to an order-of-magnitude drop in half maximal inhibitory concentration (IC_50_) (51, 52). Extrapolating this relationship, the >15 kcal/mol ΔΔ*G* enhancement observed for our top variants against KP.3/XEC points to multi-log neutralization gains, although the exact IC_50_ shift must ultimately be confirmed in functional assays. When benchmarked against literature-reported broad-spectrum nanobodies V_H_ ab6 (32) and Nanosota9 (33), R14-OPT1 achieved higher binding affinity despite targeting a more plastic epitope. This demonstrates that computational repurposing can confer breadth even on epitopes traditionally considered mutationally vulnerable.

Despite providing comprehensive analysis, this study has several limitations that need to be considered. While our pipeline predicted high-affinity nanobodies, direct comparison to clinical-stage anti-coronavirus nanobodies was constrained by their absence in late development pipelines (33). Furthermore, experimental validation of the identified mutations and the exploration of additional mutation combinations remains essential. And our strategy holds promises for extension beyond SARS-CoV-2. Residues that are critical for the development of broad-spectrum nanobodies are conserved across betacoronaviruses, such as SARS-CoV and MERS-CoV. Future work should leverage our pipeline to engineer pan-sarbecovirus therapeutics, potentially integrated with machine learning approaches to identify key residues and optimize nanobody interactions against a broader range of viral variants.

## 5 Conclusion

This study confirmed that the Q493E mutation is a key driver of immune evasion in KP.3/XEC through disruption of electrostatic nanobody-RBD interactions. To overcome this challenge, we developed a streamlined computational pipeline integrating high-throughput mutagenesis of CDRs and hotspot residues, protein-protein docking, and MD simulations. Using this approach, we repurposed four nanobodies (R14, DL4, VH ab6, and Nanosota9) to target the Omicron RBD. Notably, MD simulations validated the best-performing R14 optimized combination, R14-OPT1 systems showed a 62.6% binding energy improvement against JN.1, with a consistent binding across variants (<15% affinity variation). These results collectively demonstrate our pipeline’s capacity to significantly improve nanobody binding affinity (achieving >60% enhancement for key variants) while maintaining broad neutralization capacity. Our pipeline provides critical insights into the interactions between nanobodies and evolving viral variants, supporting the potential use of existing nanobodies as therapeutic agents.

## Data Availability

The original contributions presented in the study are included in the article/**Supplementary Material**. Further inquiries can be directed to the corresponding author.
