# Supplementary material for: From immune evasion to broad in silico binding: computational optimization of SARS-CoV-2 RBD-targeting nanobody
Source: Front Immunol. 2025 Aug 14;16:1637955. doi: 10.3389/fimmu.2025.1637955 (PMC12391910; doi:10.3389/fimmu.2025.1637955)
Supplement: Supplementary file 1 [file Table1.docx]

Supplementary Material

### Figure S1 The values of the root mean square deviation (RMSD) of the Nb-RBD complexes. (A-D) RMSD profiles for the R14-RBD, 4-RBD, V_H_ ab6-RBD and Nanosota9-RBD complexes, respectively. Each figure includes four RBDs: WT (gray), BA.2 (light pink), JN.1 (dark pink), and KP.3/XEC (red).

**
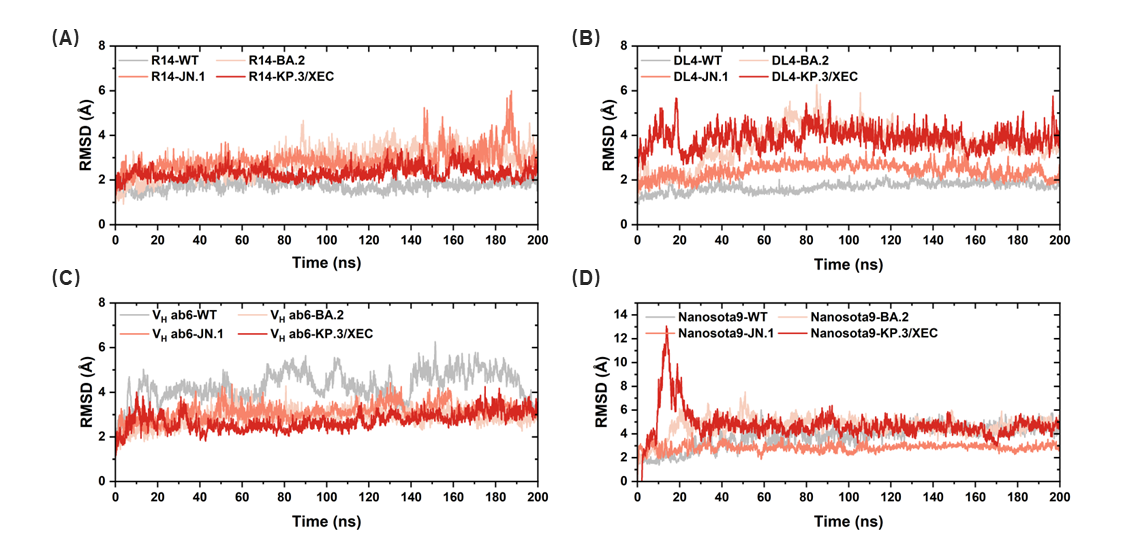
**

### Figure S2 Contour maps of free energy landscape (FEL) as a function of RMSD value and radius of gyration (Rg). (A-D) The FEL of R14 complexed with RBD (WT, BA.2, JN.1, and KP.3/XEC). (E-H) (**I**-**L**) and (M-P) The FEL of DL4, V_H_ ab6 and Nanosota9 complexed with RBD (WT, BA.2, JN.1, and KP.3/XEC), respectively.


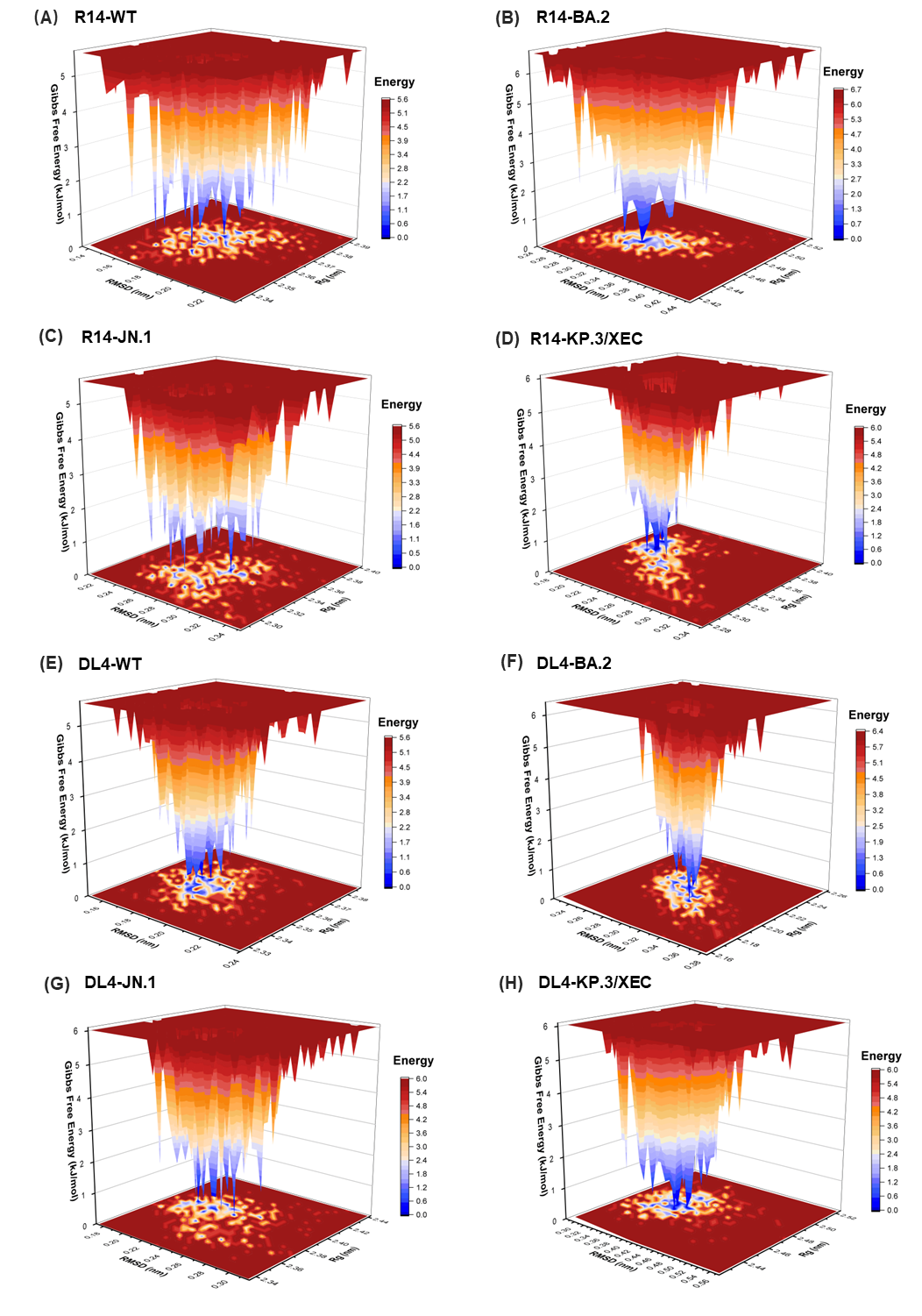


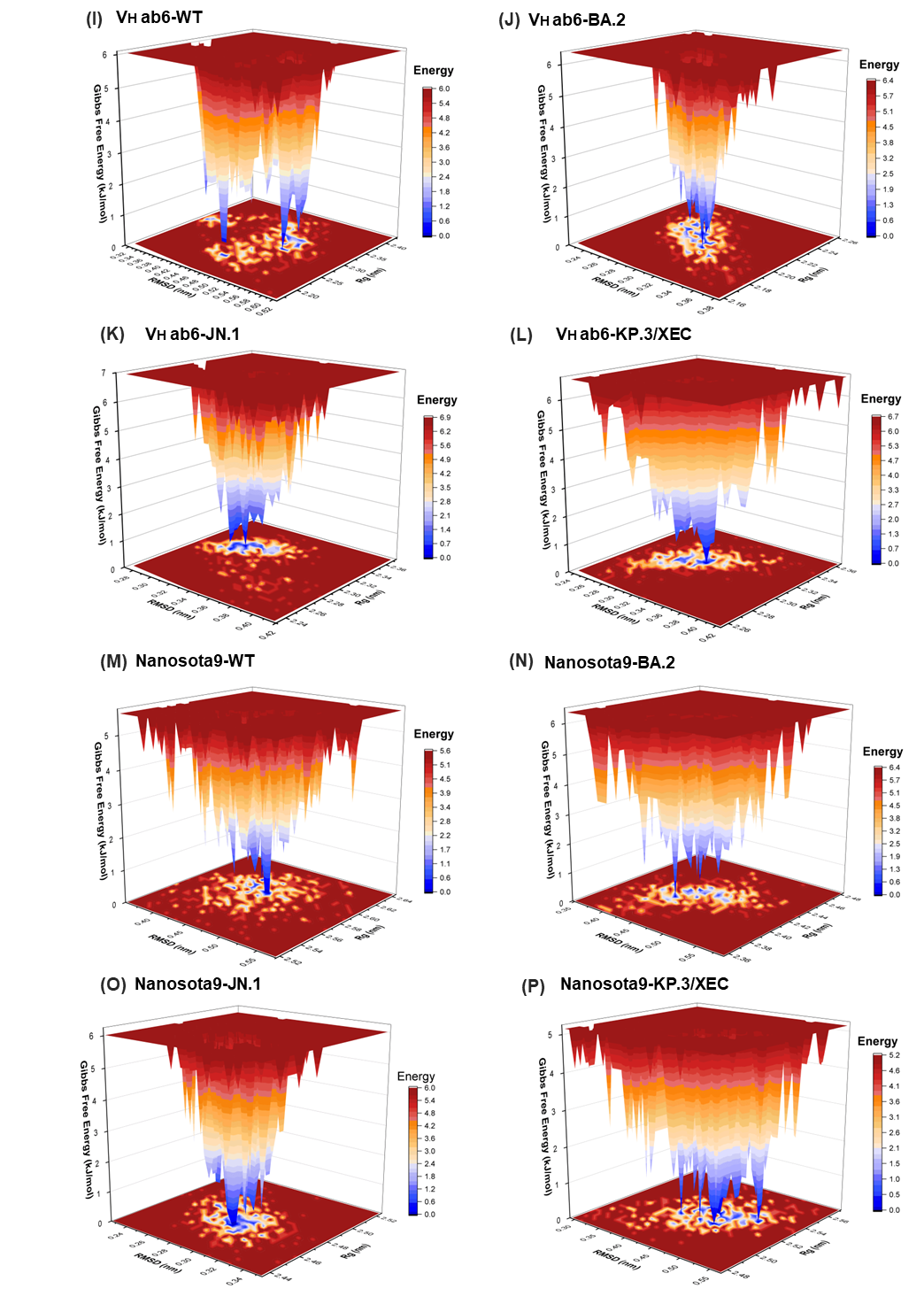


### Figure S3 Hydrogen bond analysis for the Nb-RBD systems. (A) Number and occupancy of hydrogen bonds formed at the R14-RBD interaction interface, including WT, BA.2, JN.1, and KP.3/XEC variants. (B-C) Stable hydrogen bonds (occupancy ≥70%) in the R14-JN. 1 and R14-KP. 3/XEC complex, marked with yellow dashed lines. (D-L) Similar analyses were performed for DL4, V_H_ ab6, and Nanosota9 nanobodies, as shown in panels D-F, G-I, and J-L, respectively.


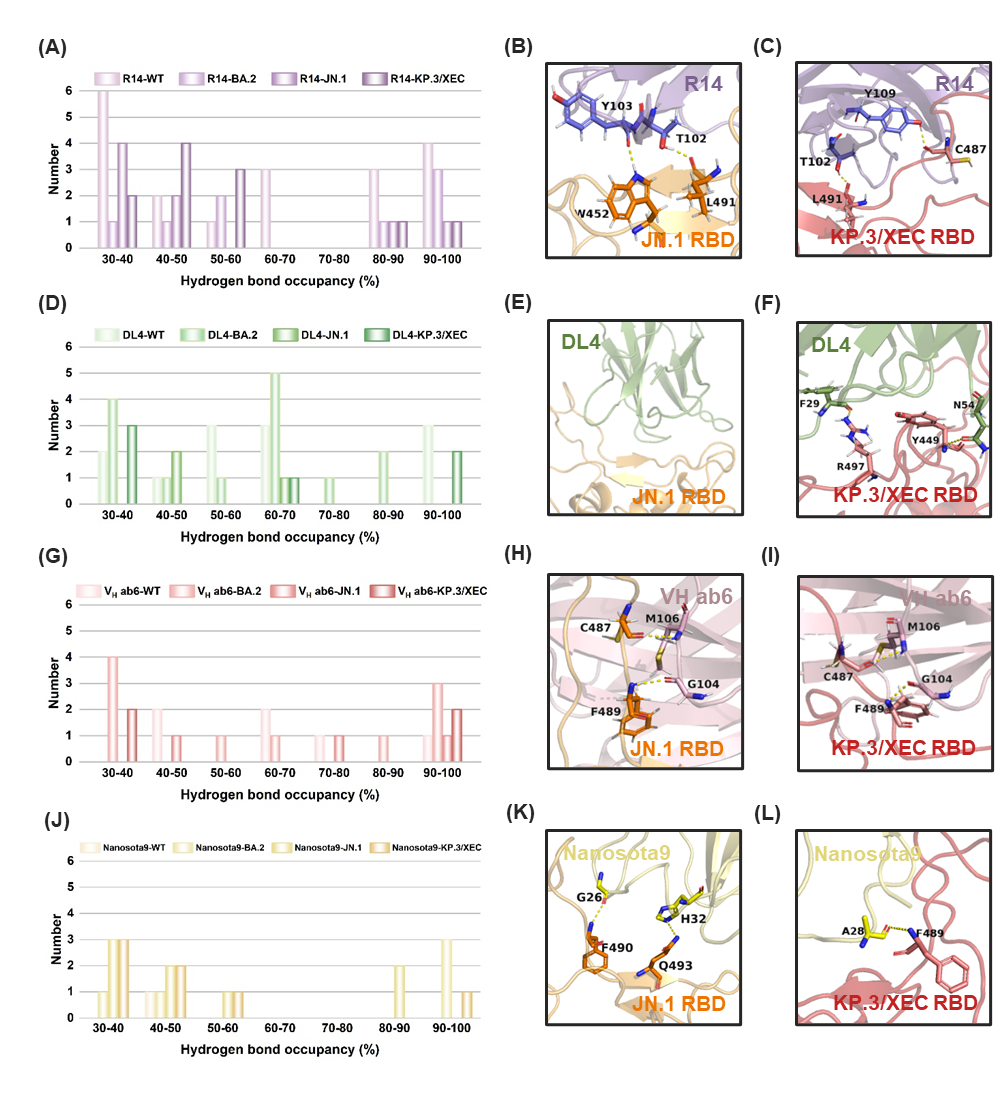


### Figure S4 Binding free energy contributions of key residues in RBD. Hotspot residues for (A) R14-JN.1, (B) R14-KP.3/XEC, (C) DL4-JN.1, (D) DL4-KP.3/XEC, (E) V_H_ ab6-JN.1, (F) V_H_ ab6-KP.3/XEC, (G) Nanosota9-JN.1and (H) Nanosota9-KP.3/XEC complexes. The energy difference between the mutant system and the WT system (ΔΔ*G_Var-WT_*) is projected on the protein structure and residues with |Δ*G_Var-WT_*| ≥ 2 (kcal/mol) are labeled in the figures.

**
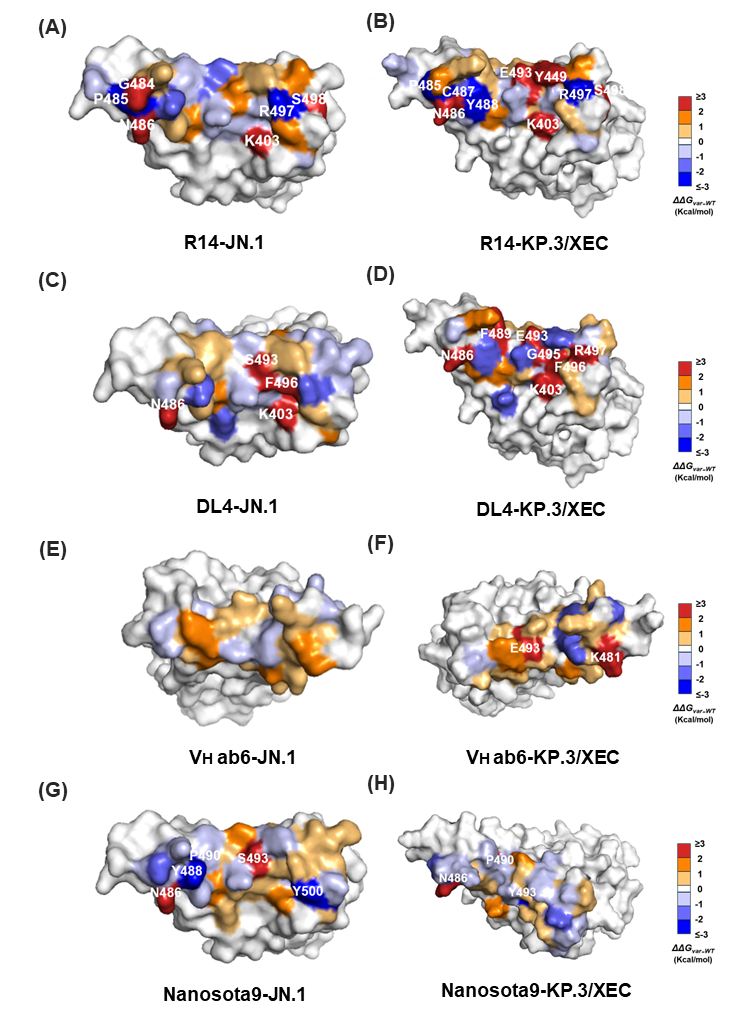
**

### Figure S5 Heat maps illustrating the *∆∆G* values for a subset of residue substitutions predicted by DDMut-PPI single-point mutations (ΔΔ*G*=Δ*G_WT_*-Δ*G_mutant_*). (A-C) Mutation heat maps for R14, DL4, and VH ab6, respectively. (D-E) Mutation heat maps for Nanosota9.


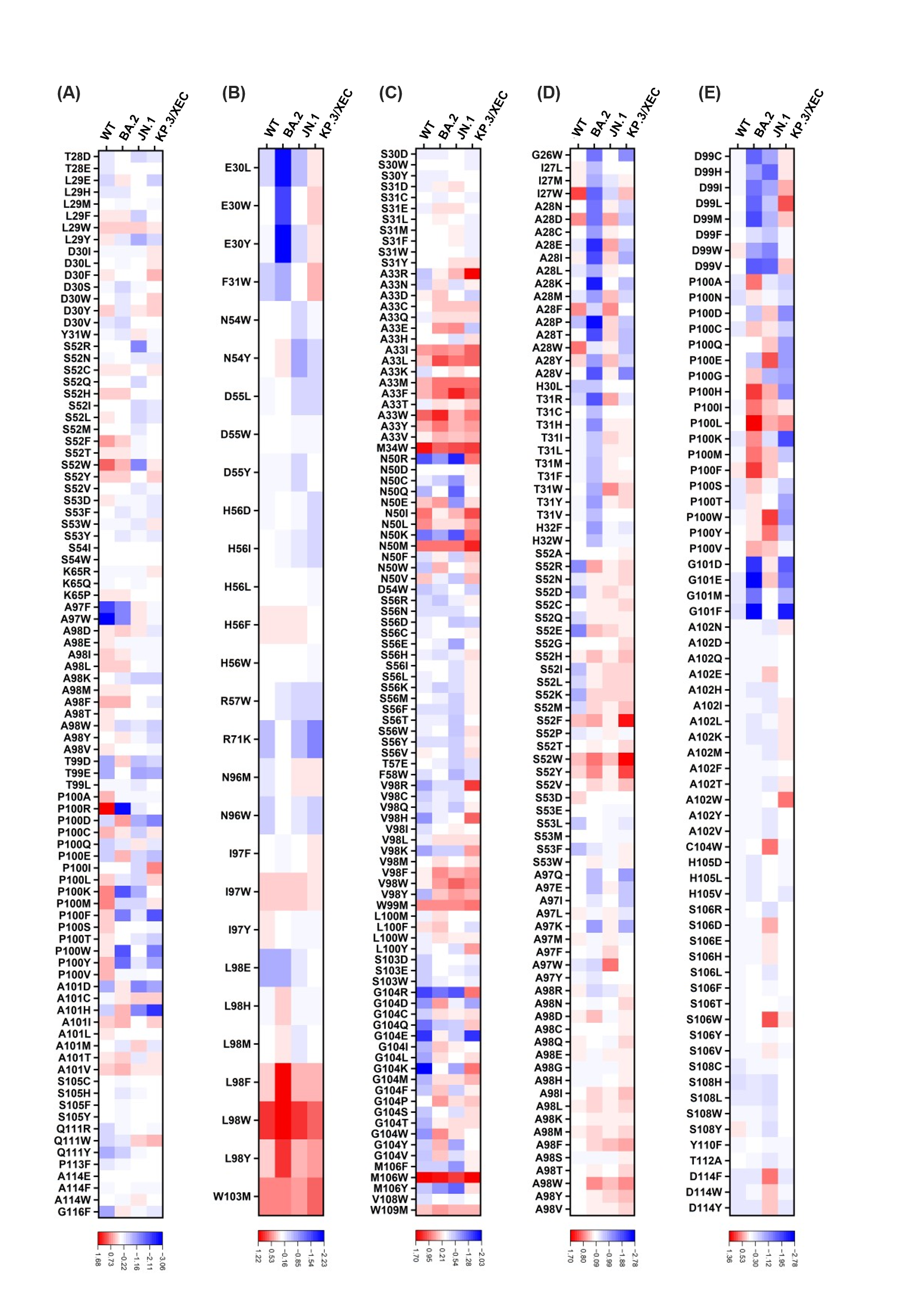


### Figure S6 The values of the RMSD of the backbone atoms of original and optimized R14-RBD complexes involved in the molecular dynamics simulation. (A-D) RMSD profiles for the R14-ORI-RBD, R14-OPT1-RBD, R14-OPT2-RBD, and R14-OPT3-RBD complexes, respectively. Each figure includes four RBDs: WT (gray), BA.2 (light pink), JN.1 (dark pink), and KP.3/XEC (red), represented by different colored lines.


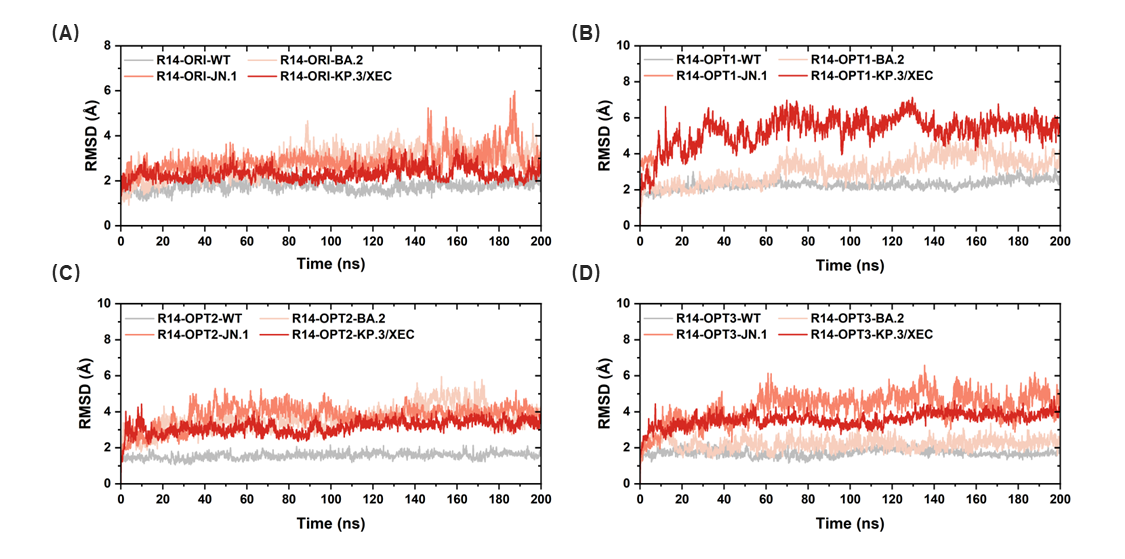


| Nanobody | SARS-CoV-2 variant | MM/PBSA  *ΔG_bind_* (kcal/mol) | MM/GBSA *ΔG_bind_* (kcal/mol) |
| --- | --- | --- | --- |
| R14 | WT | -82.35 | -90.07 |
|  | BA.2 | -60.2 | -62.39 |
|  | JN.1 | -47.3 | -46.03 |
|  | KP.3/XEC | -45.41 | -44.54 |
| DL4 | WT | -64.65 | -75.78 |
|  | BA.2 | -70.71 | -81.44 |
|  | JN.1 | -35.71 | -35.31 |
|  | KP.3/XEC | -24.8 | -19.98 |
| V_H_ ab6 | WT | -63.66 | -60.80 |
|  | BA.2 | -68.73 | -66.99 |
|  | JN.1 | -51.23 | -44.44 |
|  | KP.3/XEC | -49.7 | -44.35 |
| Nanosota9 | WT | -41.29 | -29.87 |
|  | BA.2 | -58.94 | -50.72 |
|  | JN.1 | -49.45 | -34.56 |
|  | KP.3/XEC | -37.3 | -32.55 |
| R14-OPT1 | WT | -67.04 | -71.89 |
|  | BA.2 | -70.56 | -65.18 |
|  | JN.1 | -76.88 | -73.62 |
|  | KP.3/XEC | -64.49 | -56.07 |
| R14-OPT2 | WT | -67.9 | -66.91 |
|  | BA.2 | -72.8 | -78.96 |
|  | JN.1 | -62.86 | -68.16 |
|  | KP.3/XEC | -78.09 | -81.09 |
| R14-OPT3 | WT | -73.35 | 78.20 |
|  | BA.2 | -79.47 | -86.22 |
|  | JN.1 | -55.69 | -54.42 |
|  | KP.3/XEC | -52.32 | -54.30 |

### Table S1 The list of the binding affinity of the Nb-RBD and Nb-OPT-RBD complexes

### Table S2 The list of hotspot residues of Nbs

| Nanobody | Hotspot Residue Number | Energy Contribution (kcal/mol)  **ΔΔ*G*** *_KP3/XEC_* ***_- WT_*** |
| --- | --- | --- |
| R14 | 65 | 1.21 |
|  | 101 | 1.07 |
| DL4 | 30 | 1.76 |
|  | 71 | 3.61 |
| V_H_ ab6 | 35 | 1.76 |
|  | 54 | 3.61 |
| Nanosota9 | 72 | 1.90 |

### Table S3 The list of CDRs of Nbs

| Nanobody | CDR Residue Number |
| --- | --- |
| R14 | CDR1: 26-32 |
|  | CDR2: 52-56 |
|  | CDR3: 97-116 |
| DL4 | CDR1: 26-32 |
|  | CDR2: 51-58 |
|  | CDR3: 96-103 |
| V_H_ ab6 | CDR1: 26-35 |
|  | CDR2: 50-58 |
|  | CDR3: 97-109 |
| Nanosota9 | CDR1: 26-32 |
|  | CDR2: 52-56 |
|  | CDR3: 97-116 |

### Table S4 The list of selected mutations for the R14, DL4, VH ab6, and Nanosota9 nanobodies identified after *in silico* affinity maturation using DDMut-PPI single mutations.

| Nanobody | Mutation | Predict binding affinity by DDMut-PPI (kcal/mol) | | | |
| --- | --- | --- | --- | --- | --- |
|  |  | WT | BA.2 | JN.1 | KP.3/XEC |
| R14 | L29W | 0.276 | 0.282 | 0.389 | 0.194 |
|  | S52C | 0.131 | 0.186 | 0.048 | 0.121 |
|  | A101L | 0.158 | 0.011 | 0.067 | 0.034 |
|  | A101V | 0.362 | 0.525 | 0.234 | 0.23 |
| DL4 | I97W | 0.265 | 0.204 | 0.282 | 0.088 |
|  | L98F | 0.28 | 1.119 | 0.342 | 0.362 |
|  | L98W | 0.937 | 1.215 | 0.978 | 0.728 |
|  | L98Y | 0.208 | 0.876 | 0.34 | 0.458 |
|  | W103M | 0.629 | 0.592 | 0.475 | 0.674 |
| V_H_ ab6 | A33C | 0.058 | 0.446 | 0.393 | 0.338 |
|  | A33I | 0.593 | 0.829 | 0.634 | 1.006 |
|  | A33L | 0.399 | 1.1 | 0.944 | 1.024 |
|  | A33M | 0.504 | 0.844 | 0.906 | 0.867 |
|  | A33F | 0.466 | 0.885 | 1.328 | 1.024 |
|  | A33W | 1.006 | 1.36 | 0.52 | 0.938 |
|  | A33Y | 0.482 | 0.95 | 0.565 | 0.649 |
|  | A33V | 0.161 | 0.541 | 0.373 | 0.534 |
|  | M34W | 1.483 | 0.996 | 1.132 | 1.231 |
|  | N50I | 0.893 | 0.169 | 0.484 | 1.143 |
|  | N50L | 0.728 | 0.261 | 0.212 | 0.6 |
|  | N50M | 0.901 | 0.841 | 0.853 | 1.337 |
|  | V98F | 0.121 | 0.714 | 0.552 | 0.59 |
|  | V98W | 0.17 | 0.66 | 0.959 | 0.773 |
|  | W99M | 0.742 | 0.762 | 0.822 | 0.912 |
|  | G104P | 0.082 | 0.599 | 0.27 | 0.388 |
|  | M106W | 1.633 | 1.53 | 1.251 | 1.653 |
|  | W109M | 0.4 | 0.591 | 0.5 | 0.498 |
| Nanosota9 | S52C | 0.007 | 0.16 | 0.15 | 0.325 |
|  | S52H | 0.072 | 0.361 | 0.117 | 0.435 |
|  | S52F | 0.489 | 0.577 | 0.119 | 1.489 |
|  | S52W | 0.504 | 0.915 | 0.388 | 1.694 |
|  | S52Y | 0.299 | 0.767 | 0.103 | 1.249 |
|  | A98L | 0.08 | 0.26 | 0.112 | 0.333 |
|  | A98M | 0.173 | 0.292 | 0.173 | 0.303 |
|  | P100L | 0.061 | 1.341 | 0.482 | 0.646 |
|  | P100F | 0.166 | 1.068 | 0.382 | 0.012 |

### Table S5 The list of selected mutations for the R14, DL4, V_H_ ab6, and Nanosota9 nanobodies identified after *in silico* affinity maturation using DDMut-PPI multiple mutations.

| Nanobody | Mutation | Predict binding affinity by DDMut-PPI (kcal/mol) | | | |
| --- | --- | --- | --- | --- | --- |
|  |  | WT | BA.2 | JN.1 | KP.3/XEC |
| R14 | L29W; S52C; A101V | 0.89 | 1.26 | 0.46 | 0.76 |
|  | L29W; A101V | 0.6 | 1.21 | 0.76 | 0.64 |
|  | L29W; S52C; A101L | 0.65 | 1.06 | 0.81 | 0.61 |
| DL4 | I97W; L98W; W103M | 1.47 | 2.05 | 0.84 | 1.56 |
|  | L98W; W103M | 1.53 | 1.27 | 0.72 | 1.16 |
|  | I97W; L98W | 1.05 | 1.85 | 0.94 | 0.73 |
| V_H_ ab6 | A33L; M34W; N50M; V98W; W99M; G104P; M106W; W109M | 7.97 | 7.64 | 8.05 | 8.94 |
|  | A33M; M34W; N50M; V98F; W99M; M106W; W109M | 7.38 | 7.49 | 8.79 | 8.44 |
|  | A33Y; M34W; N50M; V98W; W99M; M106W; W109M | 7.26 | 8.74 | 7.73 | 8.32 |
|  | A33F; M34W; N50M; V98W; W99M; M106W | 8.02 | 7.72 | 7.31 | 8.98 |
|  | A33Y; M34W; N50L; V98F; W99M; G104P; M106W; W109M | 7.5 | 7.96 | 7.04 | 8.51 |
|  | A33L; M34W; N50M; V98F; W99M; G104P; M106W; W109M | 7.12 | 7.2 | 7.67 | 8.97 |
|  | A33L; M34W; N50M; V98W; W99M; M106W; W109M | 7.25 | 7.83 | 7.22 | 8.61 |
|  | A33F; M34W; N50M; V98W; W99M; M106W; W109M | 7.55 | 7.75 | 7.06 | 8.41 |
|  | A33F; M34W; N50I; V98F; G104P; M106W; W109M | 7.85 | 7.82 | 6.78 | 8 |
|  | A33W; M34W; N50M; V98F; G104P; M106W | 7.73 | 8.53 | 6.94 | 7.01 |
|  | A33L; M34W; N50L; V98W; W99M; G104P; M106W; W109M | 7.11 | 8.13 | 6.91 | 7.86 |
| Nanosota9 | S52W; A98M; P100L | 0.78 | 2.24 | 1.18 | 2.98 |
|  | S52F; A98M; P100L | 1.09 | 1.99 | 0.91 | 2.46 |
|  | S52W; A98L; P100F | 1.07 | 1.89 | 1.6 | 1.86 |
|  | S52F; A98L; P100L | 0.97 | 2.14 | 0.86 | 2.45 |
|  | S52W; A98M; P100F | 1.31 | 2.33 | 1 | 1.63 |
|  | S52Y; A98M; P100L | 0.89 | 2.21 | 0.71 | 2.35 |
|  | S52F; A98M; P100F | 0.87 | 1.8 | 1.49 | 2 |
|  | S52Y; A98M; P100F | 0.58 | 2.02 | 1.52 | 1.96 |
|  | S52F; A98L; P100F | 0.6 | 1.75 | 1.43 | 2.25 |

### Table S6 The list of selected mutations for R14 identified after *in silico* affinity maturation using DDMut-PPI multiple mutations. Docking and energy analysis results are provided for each mutation.

| Nanobody | Mutation | Predict binding affinity by DDMut-PPI (kcal/mol) | | HDOCK | | MM/PBSA in 200 ns MD  (kcal/mol) |
| --- | --- | --- | --- | --- | --- | --- |
|  |  |  |  | Docking score | Confidence Score |  |
| R14 | L29W;S52C; A101V | WT | 0.89 | -446.08 | 0.9973 | -67.04 |
|  |  | BA.2 | 1.26 | -509.88 | 0.9993 | -70.56 |
|  |  | JN.1 | 0.46 | -490.12 | 0.9989 | -76.88 |
|  |  | KP.3/XEC | 0.76 | -447.54 | 0.9974 | -64.49 |
|  | L29W; A101V | WT | 0.6 | -461.52 | 0.9980 | -67.9 |
|  |  | BA.2 | 1.21 | -542.71 | 0.9996 | -72.8 |
|  |  | JN.1 | 0.76 | -483.75 | 0.9987 | -62.86 |
|  |  | KP.3/XEC | 0.64 | -445.41 | 0.9973 | -78.09 |
|  | L29W;S52C; A101L | WT | 0.65 | -472.44 | 0.9984 | -73.35 |
|  |  | BA.2 | 1.06 | -534.07 | 0.9995 | -79.47 |
|  |  | JN.1 | 0.81 | -473.58 | 0.9985 | -55.69 |
|  |  | KP.3/XEC | 0.61 | -406.00 | 0.9941 | -52.32 |

### Table S7 The list of R14 and R14-OPT protein sequence

| Nanobody | Protein Sequence |
| --- | --- |
| R14 | QVQLQESGGGLVQPGGSLRLSCAVSGFTLDYYAIGWFRQAPGKEREGVSCISSSDGSTSYADSVKGRFTISRDNAKNTVYLQMNSLKPEDTALYYCAATPATYYSGRYYYQCPAGGMDYWGQGTQVTVSS |
| R14-OPT1 | QVQLQESGGGLVQPGGSLRLSCAVSGFTWDYYAIGWFRQAPGKEREGVSCICSSDGSTSYADSVKGRFTISRDNAKNTVYLQMNSLKPEDTALYYCAATPVTYYSGRYYYQCPAGGMDYWGQGTQVTVSS |
| R14-OPT2 | QVQLQESGGGLVQPGGSLRLSCAVSGFTWDYYAIGWFRQAPGKEREGVSCISSSDGSTSYADSVKGRFTISRDNAKNTVYLQMNSLKPEDTALYYCAATPVTYYSGRYYYQCPAGGMDYWGQGTQVTVSS |
| R14-OPT3 | QVQLQESGGGLVQPGGSLRLSCAVSGFTWDYYAIGWFRQAPGKEREGVSCICSSDGSTSYADSVKGRFTISRDNAKNTVYLQMNSLKPEDTALYYCAATPLTYYSGRYYYQCPAGGMDYWGQGTQVTVSS |
